# Variant Splicing and Influence of Ionizing Radiation on Human Endogenous Retrovirus K (HERV-K) Transcripts in Cancer Cell Lines

**DOI:** 10.1371/journal.pone.0076472

**Published:** 2013-10-18

**Authors:** Lorenzo Agoni, Jack Lenz, Chandan Guha

**Affiliations:** 1 Department of Pathology, Albert Einstein College of Medicine, Bronx, New York, United States of America; 2 Department of Genetics, Albert Einstein College of Medicine, Bronx, New York, United States of America; 3 Department of Radiation Oncology, Albert Einstein College of Medicine, Montefiore Medical Center, Bronx, New York, United States of America; Plymouth University, United Kingdom

## Abstract

Human endogenous retrovirus K (HERV-K) is the most intact retrovirus in the human genome. There are multiple full-length or near full-length HERV-K proviruses in it. To analyze which HERV-K proviruses give rise to viral transcripts in cancer cell lines and to test whether ionizing radiation can alter the levels of HERV-K transcripts, RT-PCR studies were undertaken using multiple human cancer cell lines. Primers from several positions in the viral genome were used and included pairs designed to cross splice junctions in viral RNAs. In the absence of ionizing radiation, transcripts were detected from multiple HERV-K proviruses in cell lines from human prostate, cervical, head and neck, or breast cancers, and the proviruses from which the transcripts originated varied among the different lines. Only one of 13 cell lines tested (cervical cancer line C33A) failed to show HERV-K transcripts. Spliced RNAs detected included viral RNAs spliced as expected at the conventional viral splice sites, plus several alternatively spliced RNAs. Alternatively spliced transcripts arose from specific proviruses, and were detected in most of the cell lines used. Quantitative RT-PCR was performed to assess the effects of ionizing radiation. These analyses showed that HERV-K transcripts were elevated in four of twelve lines tested, specifically all three prostate cancer lines used and one breast cancer line. The increases were transient, peaking at 24 hours following a single dose of gamma-irradiation that ranged from 2.5 to 20 Gy, and returning to baseline levels by 72 hours. In summary, these studies showed that ionizing radiation can affect the levels of HERV-K transcripts in cells, and these effects vary among different cells. The changes in HERV-K transcript levels might affect multiple biological processes in cells, and future studies of the effects of ionizing radiation on HERV-K are worth pursuing.

## Introduction

The effects of ionizing radiation (IR) on endogenous retroviruses (ERVs) are largely unknown. The DNA genomes of ERVs are integrated into the genome of their host species as a result of infections of germline cells over evolutionary time. To date, the effects of ionizing radiation on ERVs have been explored only in mice [1], where it has been known for decades that ionizing radiation could reactivate mouse endogenous retroviruses (MERVs) [2]–[4]. Ionizing radiation also affects immune responses to cancer cells, and one study detected CD8+ T-cells reactive toward a MERV antigen in a murine, irradiated colon carcinoma model [5]. Thus it is plausible that effects of ionizing radiation on endogenous retrovirus expression might have consequences for immune responses toward tumor cells.

Ionizing radiation has been reported to increase the transcriptional activity of many viruses including CMV [6], [7], HPV [8] and HIV [9], [10] in infected cells. The molecular mechanisms are still largely uncharacterized, although for HIV, IR has been reported to increase transcription through LTR promoter activation [9], [10]. At the clinical level, studies have shown an unexpectedly high reactivation rate of latent HBV infection in hepatocellular carcinoma after radiotherapy [11]. In HPV infected cells, increased production of E6/E7 transcripts and proteins has been shown after γ-irradiation [8]. At a clinical level, increased specific cytotoxic lymphocytes (CTL) responses against HPV E6/E7 antigens have been detected in cervical cancer patients after radiotherapy [12]. In addition to effects on levels of virus expression, ionizing radiation is known to modulate immune responses against cancer [13]–[15].

Endogenous retroviruses are present in the genome of every cell of an individual and can be targets for immune responses such as those against MERVs [16]. Both T- and B-lymphocyte responses have also been observed against human endogenous retroviruses (HERVs) [17]–[21]. Most notably, immune responses have been observed against the most recently acquired set of retroviruses in the human genome, HERV-K [18], [19], [22], [23]. HERVs exist in the human genome in the form of integrated retroviral DNA genomes called proviruses. Collectively they have been estimated to comprise about 8% of the human genome [24]. In the absence of selection pressure on the host to maintain these proviruses in an intact form, mutations inevitably accumulate in them over evolutionary time thereby disrupting viral genomic components and inactivating viral infectivity. The HML2 subtype of HERV-K is the only retrovirus to have entered the genome of the human lineage since the divergence of the human and chimpanzee lineages occurred about 6 million years ago [25]–[31]. Because it is the most recently inserted, HERV-K HML2 constitutes the most intact set of retroviruses in the human genome [25], [32]–[39]. Despite that, no individual HERV-K proviral locus is known that is fully functional and able to produce infectious virions, although recombination among the loci existing today in the human genome could restore viral infectivity [37]. Many of the HERV-K proviruses are sufficiently intact to encode proteins that may retain some functionality [25], [35], [36], [38], [39], and expression of these proteins has been suggested to correlate with diseases including cancer [40]–[44].

HERV-K is transcribed to various extents in several human diseases including cancer [20], [45]–[48], HIV infection [18], [49]–[51] and autoimmune disorders [52], [53]. Individual HERV-K loci in the human genome differ in the integrity of specific open reading frames, functionality of the encoded proteins, the integrity of cis-acting elements, and possibly in the capacity of DNA sequences flanking the viral DNA to affect transcription. They vary from approximately 97 to over 99% identical in pairwise comparisons to each other, and many of the differences among them are single base pair changes. Therefore to identify the specific HERV-K loci expressed in a human sample and to understand thoroughly the biological role of HERV-K in human diseases, if any, it is necessary to include analysis of viral transcripts at the single nucleotide level in any study of them [54]. We previously detected HERV-K transcripts in prostate cancer cell lines and observed that some of the transcripts were spliced at unusual positions in the viral genome [55]. The detection of variant splicing of HERV-K transcripts in prostate cancer lines was unexpected. In this report, the nucleotide based analysis of specific HERV-K loci that are transcribed and the possibility of unusual splicing variants were extended to additional types of cancer cell lines. We also tested the hypothesis that ionizing radiation may alter the levels of human endogenous retrovirus K transcripts. HERV-K proteins are known to be targets for both T- and B- lymphocyte responses [17]–[21], [46]. As a first step toward determining whether HERV-K antigens might be subject to irradiation-induced modulation and perhaps constitute candidate targets for anti-tumor, T-cell based immunotherapy for cancer following administration of ionizing radiation, we investigated whether ionizing radiation affects the levels of HERV-K transcripts in various cancer cell lines.

## Results

### HERV-K Transcription in Cancer Cell Lines

**Figure 1:**
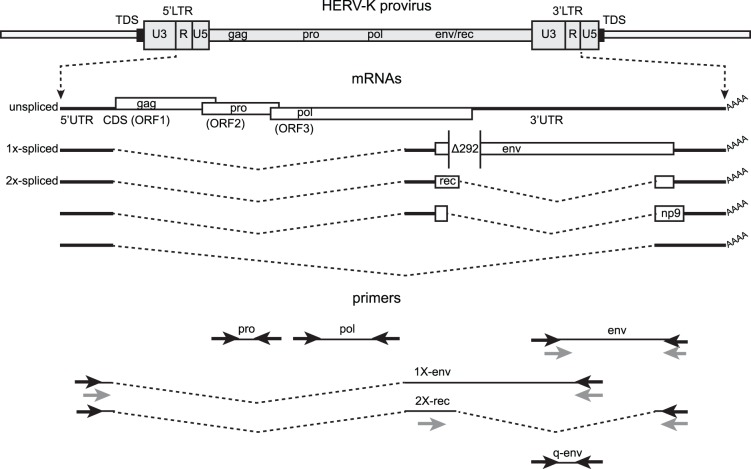
Structure of the HERV-K genome and spliced mRNAs showing the primers used for reverse transcription and PCR. A genetic map of a HERV-K provirus (gray) inserted into flanking host genome sequences is shown with a 5 or 6 bp target duplicated sequence (TDS) indicated as black boxes. The unspliced primary viral transcript, singly-spliced *env* mRNA and doubly-spliced *rec* and *np9* mRNAs are shown below the viral genome, along with the singly spliced RNA [56] that is not known to encode any protein. 3′ poly(A) tails are indicated (AAAA). The 292 nucleotide deletion of type 1 HERV-K proviruses spanning the pol-env junction is indicated (Δ292). Type 2 HERV-K proviruses and their transcripts contain these nucleotides. Positions of the PCR primer pairs are shown at the bottom as black arrows with the names by which they are identified throughout the paper. The gray arrows identify primers used for nested PCR. The dashed, angled line shows the excised intronic sequences that the 1X-env and 2X-rec primer pairs were designed to cross. The primer pair used for quantitative RT-PCR is also shown (q-env).

**Figure 2:**
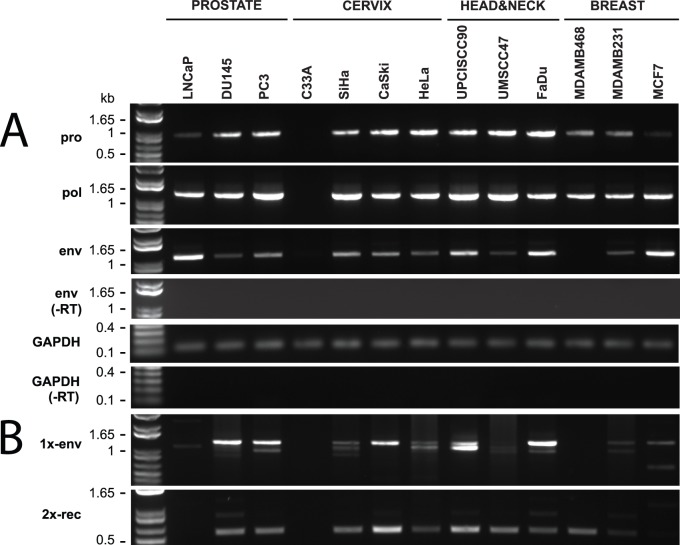
Detection of HERV-K transcripts in 13 cancer cell lines. RT-PCR was performed to detect viral transcripts at five different positions in HERV-K genome, two of which across splicing junctions to detect RNAs spliced at the conventional env mRNA splice junction (1x-env) and the rec mRNA splice junctions. GAPDH RT-PCR was performed simultaneously and served as a positive control for RNA integrity and as loading comparison. The products were resolved by agarose gel electrophoresis. Genomic positions of the primers used are shown in Fig. 1. Parallel controls were performed without reverse transcriptase (−RT) as controls to exclude DNA contamination. DNA size markers are shown on the left.

HERV-K transcription has been reported to occur in human cancer. However, the response of HERV-K to ionizing radiation has never been tested in cancer or normal cells. To verify HERV-K transcription and establish a reference baseline for experiments with IR, we first performed reverse transcriptase-PCR (RT-PCR) on RNA isolated from 13 cancer cell lines from cancer sites commonly treated with radiotherapy: prostate, uterine cervix, head & neck, and breast. RT-PCRs were performed using primer pairs from multiple parts of the viral genome shown in Figure 1. Primers from the viral protease (*pro*), polymerase (*pol*) were capable of detecting unspliced viral RNA, and envelope (*env*) gene primers were able to detect both unspliced and singly spliced env mRNAs. The expected size products were observed in 12 cell lines out of the 13 tested for the *pro* and *pol* amplicons and in 11 cell lines for the *env* amplicon (Fig. 2A). Parallel RT-PCRs were performed with primers for the *GAPDH* gene to verify accurate comparable loading of RNA into all samples and loading of gels. Controls without reverse transcriptase verified that products were generated from RNA templates and not from potentially contaminating genomic DNA (Fig. 2).

**Figure 3:**
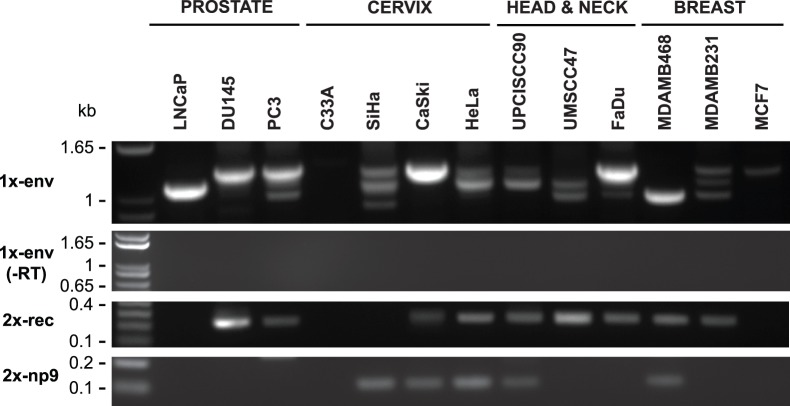
Detection of HERV-K transcripts in 13 cancer cell lines. Nested PCR was performed on RT-PCR products shown in Fig. 2 to detect viral transcripts at three different positions in HERV-K genome. Two of RT-PCRs cross splicing junctions to detect RNAs spliced at the conventional env mRNA splice junction (1x-env) and rec and np9 mRNA (2X-rec, 2X-np9) splice junctions. The products were resolved by electrophoresis. Genomic positions of the primers used are shown in Fig. 1. Parallel controls were performed without reverse transcriptase (-RT) as controls to exclude DNA contamination. DNA size markers are shown on the left.

These data confirmed that HERV-K transcripts were present in most of the human cancer cell lines tested. There were a few instances where HERV-K RT-PCR products were not detected (Fig. 2). One cervix cancer cell line, C33A, did not yield RT-PCR products with any of the three primer pairs, although it did give the expected product for in the *GAPDH* control amplification (Fig. 2A). One breast cancer cell line, MDA-MB-468, consistently failed to yield the *env* amplicon, although the *pro* and *pol* products were present (Fig. 2A). To increase the sensitivity, a nested RT-PCR was performed for the *env* amplicon using primers positioned as shown in Figure 1. The *env* product was detected in the MDA-MB-468 breast cancer line, as it was in 11 other lines (Fig. 3A). However, it still was undetectable in C33A cervical cancer cells, indicating that the unspliced and singly spliced HERV-K RNAs were not present at detectable levels in this cell line. Parallel amplification reactions were performed without reverse transcriptase and were uniformly negative, thus showing that amplification was from RNA templates and not from any potentially contaminating genomic DNA (Fig. 3).

### Presence of HERV-K Spliced Transcripts in Cancer Cell Lines

To assess whether the HERV-K transcripts have sufficient integrity for the RNAs to be spliced, we performed RT-PCR across the two viral conventional splicing junctions. Multiple spliced HERV-K mRNAs have been characterized [40], [56]–[58] including the singly spliced mRNA encoding the Env protein, and three doubly spliced RNAs, one of which encodes Rec, and another of which encodes Np9 (Fig. 1). HERV-K HML2 proviruses exist as two different types called type 1 and type 2, depending on whether a 292 bp stretch of nucleotides is present (type 2) or deleted (type 1). In type 1 proviruses, the deletion removes the amino terminal-encoding portion of *env* and *rec*, and causes an in frame fusion of the *pol* and *env* open reading frames (Fig. 1). Conversely, in type 2 proviruses, the carboxyl terminus-encoding portion of *pol* and the that for amino termini of *env* and *rec* are fully present. The 3′ splice site (3′SS) for *env* mRNA and the first intron of *rec* mRNA is situated upstream of the 292 bp stretch, but the 5′SS for the second intron of *rec* mRNA is located within the 292 bp (Fig. 1). Thus type 1 proviruses do not generate the *rec* form of doubly spliced RNA (Fig. 1) or encode the Rec protein.

Using primers designed to detect the singly-spliced *env* mRNA (1X-env, Fig. 1) and the two splice junctions in doubly-spliced *rec* or *np9* mRNAs (called 2X-rec, Fig. 1), 11 out of 13 cell lines were found to be positive with the 1X-*env* primers, and 10 out of 13 yielded products with the 2X-rec primers (Fig. 2B). Detection of the spliced products also provided further evidence that the RT-PCR products were derived in fact from virally encoded RNAs and not from any potentially contaminating cellular genomic DNA. The detection of multiple size products with the 1X-env primers was unexpected.

To verify that the unexpected size bands were derived from HERV-K RNAs and to achieve a better resolution of the PCR products bands on agarose gel electrophoresis, we performed nested PCRs (Figure 1). Multiple bands were again observed, and no products were detected in the absence of reverse transcriptase, thus confirming that they were derived from spliced viral RNAs (Fig. 3). PCR products were identified for 1X-env amplicon in 12 out of 13 cell lines and bands of three different sizes were detected, with the ratios of intensity of the three bands varying among the different lines. Multiple size products were also observed for the 2X-rec amplicon, including the faint band of approximately 500 bp and a prominent band of about 250 bp. Both PCR products were present in MDA-MB-468 breast cancer cells, thus showing that spliced viral RNA was present in this cell line. For C33A cervical cancer cells, neither of the nested products from spliced RNA was detected, thereby confirming the absence of detectable HERV-K transcripts in this cell line. This analysis also detected the presence of np9 transcripts in five of the lines (Fig. 3).

### Identification of Individual HERV-K Active Loci

The reason for the detection of multiple size bands in the RT-PCRs of Figures 2B and 3, particularly for the 1X-env RT-PCRs, was uncertain. One possibility was that they could reflect unique deletions or insertions that had accrued in specific HERV-K loci over evolutionary time resulting in altered size transcripts, and these particular loci were the ones transcribed in the cell lines used. Another was that the splice sites used varied among individual HERV-K loci in the human genome, thereby resulting in different size products. To distinguish between these hypotheses and to identify the specific loci from which the transcripts originated, the PCR products shown in Figure 2 were sequenced. This required analysis of the transcripts at the nucleotide level because the proviruses are so similar to each other. While individual proviruses accumulate unique mutations over evolutionary time, some of the HERV-K proviruses in the human genome are so new that they are over 99% identical, and the level of identity can vary in different parts of the viral genome. Proviruses that formed earlier in time have gradually become more divergent. Sequencing of sufficiently long PCR products can be used to deduce the specific viral loci that most closely match the transcripts, even when the differences are less than one nucleotide in 100 between two particular proviruses and RNA transcripts from them. Table 1 lists 23 of the most intact HERV-K HML2 proviruses in the human genome to which the sequences were compared [39].

**Table 1:** Polymorphisms in cDNA sequences from *pro* amplicons in 13 cancer cell lines and reference to 22 full-length or near full-length proviruses.

				Nucleotide a											
Viral genome position^b^				3018	3171	3189	3219	3262	3297	3316	3412	3519	3641	3642	3657
**Plurality** c				A	T	A	G	A	G	A	C	G	C	T	C
**Cell lines**	LNCaP			.G	.C	C	.	.	A	.G	.	.	T	.G	.
	DU145			.	.	.	.	.	.	.	.	.	.	.	.
	PC3			.	.	.	.	.	.	.	.	.	.	.	.
	C33A			n	n	n	n	n	n	n	n	n	n	n	n
	SiHa			.	.	.	.	.	.	.	.	.	.	.	.
	CaSki			.	.	.	.	.	.	.	.	.	.	.	.
	HeLa			.	.	.	.	.	.	.	.	.	.	.	.
	UPCISCC-90			.	.	.C	.	.	.	.	.	.	.	.	.
	UMSCC-47			.	.	.	.	.	.	.	.	.	.	.	.
	FaDu			.	.	.	.	.	.	.	.	.	.	.	.
	MDA-MB-468			.	.	.C	.	.	.	.	.	.	.	.	.
	MDA-MB-231			.	.	.	.	.	.	.	.	.	.	.	.
	MCF7			.G	.C	C	.	.	.A	.G	.	.	.T	.G	.
	**Virus**	**Type**	**Position**												
***Homo sapiens*** ** specific**	K101	I	chr22∶18,926,187–18,935,361	G	C	C	A	.	.	.	.	.	.	G	.
	K102	I	chr1∶155,596,457–155,605,636	.	.	.	.	.	.	.	.	.	.	.	.
	K103	I	chr10∶27,182,399–27,183,366	G	C	C	.	.	.	.	.	.	.	.	.
	K104	II	chr5∶30,486,760–30,496,205	G	C	.	.	.	A	G	T	.	.	.	.
	K106	I	chr3∶112,743,124–112,752,282	G	C	C	.	.	A	G	.	.	T	G	.
	K107	I	chr5∶156,084,717–156,093,896	G	.	.	.	.	.	.	.	.	.	.	G
	K108	II	chr7∶4,622,057–4,640,031	G	C	C	.	.	A	.	.	.	.	G	.
	K109	II	chr6∶78,426,662–78,436,083	G	.	C	.	.	.	G	.	.	T	G	.
	K113	II	chr19∶21,841,536–21,841,541	G	.	C	.	.	A	.	.	.	.	G	.
	K114	II	chr11∶101,565,794–101,575,259	G	C	C	.	.	A	.	.	.	.	.	.
	K115	II	chr8∶7,355,397–7,364,859	–	.	.	.	.	A	.	.	.	T	G	.
	K116	I	chr3∶85,280,336–185,289,515	G	C	C	.	G	.	.	T	A	.	G	.
	K117	II	chr12∶58,721,242–58,730,698	G	C	C	.	.	A	.	.	.	.	.	.
	K(I)	II	chr3∶125,609,302–125,618,439	G	C	.	A	.	A	.	T	A	.	.	.
	K4	I	chr1∶75,842,771–75,849,143	G	C	C	.	.	A	.	.	–	–	–	–
	K60	I	chr21∶19,935,621–19,940,996	G	C	.	A	G	.	.	.	A	.	.	.
**Shared with ** ***Pan* and *Gorilla***	K105	I	chrUn:gl000219∶175210–176178	G	C	.	.	G	A	.	T	A	.	.	T
	K110	I	chr1∶160,660,575–160,669,806	G	C	G	.	G	A	.	T	.	.	.	.
	K111	I	chr11∶118,591,724–118,600,883	G	C	.	.	G	A	.	T	.	.	.	.
	K112	II	chr10∶6,866,141–6,875,603	G	C	G	.	T	A	.	T	.	.	.	T
	K118	I	chr3∶101,410,737–101,419,859	G	.	G	A	G	.	G	T	A	.	C	T
	K5	I	chr4∶165,916,840–165,924,068	G	C	.	A	G	A	.	T	A	.	.	T
	K51	I	chr19∶22,757,824–22,764,561	G	C	G	.	G	A	.	T	A	.	.	T
	K50F	II	chr19∶37,597,549–37,607,066	G	C	.	A	G	A	G	T	.	.	.	.

a Notations: “.” = same as in consensus, “n” = non detectable, “.G” = primary and secondary peaks of approximately equal detection, “–” = absent in alignment.

b Position in the reference HERV-K genome (K108).

c Plurality sequence indicates the most common nucleotide at that position among amplicons from the 13 cell lines.

**Figure 4:**
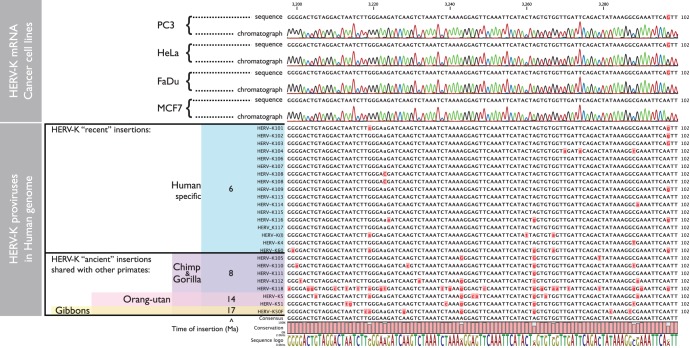
DNA sequencing chromatographs of RT-PCR products generated from RNAs isolated from four arbitrarily chosen cell lines are shown in the upper panel, one for each cancer type used in the study. In the lower panel, the HERV-K full-length proviruses used as references to identify the loci of origin of mRNA transcripts are shown. On the left, the major branches of extant hominoids are shown, along with the approximate times of their branching from the lineage leading to modern humans in millions of years ago (Ma). Proviruses that are inserted at precisely orthologous positions in humans and other hominoids, and thus are identical by descent, are indicated.

Initially, the *pro*, *pol* and *env* PCR products were directly sequenced. An example of *pro* sequences from four cell lines aligned with many of the relatively intact HERV-K proviruses in the human genome is shown in Figure 4. Comparison of the sequences showed that the HERV-K transcripts detected matched those shared by human-specific proviruses, showing that they derived at least predominantly from the subset of HERV-K HML2 proviruses that formed after the human and chimpanzee lineages diverged. At other positions, there were multiple secondary peaks in the sequencing chromatographs, indicating that the PCR products were derived from mixtures of transcripts from multiple loci. Table 1 summarizes the most predominant nucleotides (“plurality”, Table 1) at each of the positions where multiple nucleotides were unambiguously observed within the *pro* amplicons. Although, this most closely matched the provirus HERV-K102 (Table 1), it was not possible to discern by this analysis whether this provirus was in fact transcribed, because it was also plausible that the plurality sequence of a mixture of transcribed proviruses just coincidently matched this provirus. The sequence mixtures varied to some extent among the different cell lines (Table 1), and were similarly observed in the other parts of the viral genome (data not shown). While the insights that could be gained from the direct sequencing of the RT-PCR products were thus limited, the mixtures did show that multiple proviruses were transcribed among the various cancer cell lines.

To investigate more precisely which HERV-K loci were transcribed, mixtures of RT-PCR products were separated into individual sequences by cloning in plasmid vectors. This also allowed investigation of what accounted for the multiple size products (Figs. 2 and 3). The 1X-*env* RT-PCR products (Fig. 2A) were shotgun cloned into plasmid vectors. For the 1X-env products, twenty-four individual clones from each cell line were assayed by PCR screening to determine the size of the cloned amplicon. For the various samples, 4 to 13 clones were chosen for sequencing, and the results were summarized in Table 2. Clones were chosen to encompass the set of different size products in each line, and thus the number of times any sequence was obtained did not reflect its relative abundance among the HERV-K transcripts in each cell line. In addition, the extent of sequencing was limited to looking at the most common products, and it is likely that less abundantly transcribed proviruses were not detected by this analysis.

**Table 2:** Individual HERV-K loci identified by sequencing of nested RT-PCR, 1X-env RNA, spliced products.

		PROSTATE			CERVIX				HEAD&NECK			BREAST		
		LNCaP	DU145	PC3	C33A	SiHa	CaSki	HeLa	UPCISCC90	UMSCC47	FaDu	MDAMB468	MDAMB231	MCF7
K102	1x-env conventional									•				
K108	1x-env conventional		•	•		•			•	•	•		•	
K(I)	1x-env conventional	•				•		•	•	•				
K109	1x-env conventional						•							•
K117	1x-env conventional							•					•	
K106	1x-env conventional									•				
K111	1x-env alternative					•								
K102	1x-env alternative		•	•						•	•	•	•	
K(I)	1x-env alternative								•					•
K117	1x-env alternative												•	

These analyses identified a total of six different individual HERV-K loci among the 12 cell lines (Table 2). Two of these were HERV-K102 and HERV-K108 (hereafter proviruses are identified simply as K102, K108, etc.). Along with a third provirus, K(I), these were detected in multiple cell lines (Table 2). RT-PCR products were also detected from three additional proviruses, K107, K111, and K117 in one or a few cell lines each. Transcripts from multiple proviruses were detected in most of the lines. It is likely that more extensive sequencing of clones would increase the number of HERV-K loci detected. Five of the loci from which viral transcripts were detected were human-specific HERV-K proviruses, confirming that the detected transcripts largely derived from the subset of HERV-K HML2 proviruses that formed after the human and chimpanzee lineages diverged. Thus the most frequently detected HERV-K spliced *env* transcripts in the human cancer cell lines were derived from the most recently acquired subset of retroviruses in the human genome.

### Alternative Splicing of Individual HERV-K Active Loci

**Figure 5:**
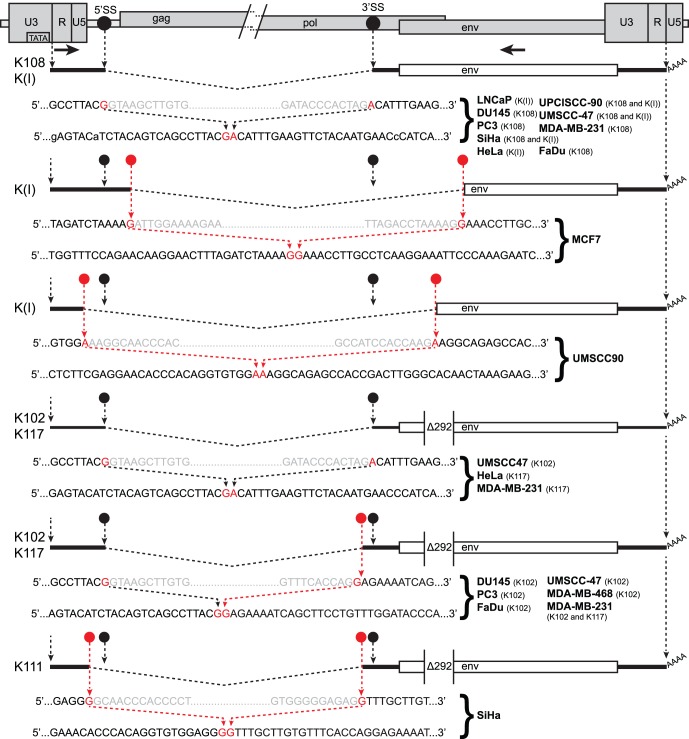
HERV-K RNA splice sites determined by sequencing of RT-PCR products. The 5′ and 3′ portions of a HERV-K provirus are shown at the top separated by/and/. 5′SS and 3′SS indicate the conventional splicing sites of HERV-K, and their positions are marked with black circles. The positions of the outer PCR primers used for the nested PCR are shown as arrows. Structures of the env spliced RT-PCR products from the cell lines and proviruses indicated are diagrammed below the viral genome. Dashed angled lines show the excised introns. Red circles show the positions of the sequences where unconventional splicing occurred. For each spliced product, the top sequence shows the inferred primary transcript sequence determined from that of the cognate genomic locus, and the bottom sequence shows the sequence of the RT-PCR product. Red nucleotides were joined in the spliced product. Gray nucleotides show the ends of the excised intronic sequences. The position of the 292 nucleotide deletion definitive of type 1 proviruses is shown.

Sequencing of the 1X-env RT-PCR products showed splicing at the expected positions within the HERV-K genome, but, unexpectedly, also showed transcripts spliced at additional sites (Figure 5). The conventional sites were detected for the type 2 proviruses, K108, K109, and K(I), and type 1 proviruses, K102 and K117 (Fig. 5). K108 is one of the most intact proviruses in the human genome having full-length open reading frames for all viral proteins [25], [35], and conventionally spliced transcripts from it were detected in seven different lines (Fig. 5). However four loci, K102, K(I), K117, and K111, showed unusual 1X-env splicing variants formed from the use of alternative 5′ and 3′ splice sites. The alternative splice sites that were used varied among the different proviruses (Fig. 5). Those that were detected (Fig. 5) in some instances matched the consensus signals for the major or minor spliceosomes, while in other instances they did not (Fig. 5). Most of alternatively spliced transcripts were from type 1 proviruses, but one of them, K(I), was from a type 2 provirus. Thus the alternative splice site usage was not solely a consequence of the absence of the 292 bp. K111 is an older provirus that formed before the divergence of the gorilla lineage from the human-chimpanzee lineage roughly 8 million years ago [25], [59], but the other proviruses from which the spliced transcripts were detected here are human-specific.

The cell lines in which the alternative 1X-env splice sites were detected are summarized in Table 2 and Figure 5. For K102, the same alternative splice sites were detected in six different cell lines, and for K117, the same sites were detected in two (Fig. 5). This suggested that the sequences of the specific transcripts (and the proviruses from which they were derived) determined alternative splice site usage. Alternatively spliced transcripts were detected in the majority of the cell lines, and in some instances, both the conventional and alternative splice sites were detected for particular proviruses (K102, K(I), and K117). It is unclear from this analysis whether the nature of the cells contributed to the splicing patterns observed. In summary, the alternative splice sites detected accounted for the different size bands detected in the RT-PCR reactions across the 1X-env splice junction (Figs. 2 and 3). However, the frequent detection of them and the variation among the different HERV-K proviruses were unexpected, and the possible molecular biological basis for them was unknown.

Sequencing of the different sized products was also undertaken for PCRs performed with the primers to detect the doubly spliced RNAs (data not shown). The less prominent, approximately 800 bp product (Fig. 2B) corresponded to conventionally spliced *rec* mRNA. Nested PCRs confirmed the presence of rec mRNA in 9 of 13 cell lines (Fig. 3). In addition, sequencing showed that the prominent smaller band in Figure 2B corresponded to RNA spliced from the 5′SS upstream of *gag* to the 3′SS downstream of the second intron in *rec* mRNA. This RNA has been described previously [57], but is not known to encode any protein.

### Ionizing Radiation (IR) Increases HERV-K Transcription in Prostate Cancer Cell Lines

Having established that HERV-K was transcribed in a variety of human cancer cell lines, we asked whether ionizing radiation could alter the levels of viral transcripts. Quantitative, RT-PCR (qRT-PCR) was performed on RNA isolated from the 12 cell lines that showed HERV-K transcription. Cells at 50% confluence were irradiated in a single exposure to a ^137^Cs source. Doses of γ-irradiation were varied at 0, 2.5, 5, 10 or 20 Gy, and cells at each dose were collected 1, 3, 8, 24, and 72 hours after irradiation. The primer pair used was designed in *env*, downstream of the 292 nucleotide deletion, so that it detected both unspliced RNA and singly-spliced env mRNA (Fig. 1). Each experiment was performed three separate times, and three RT-PCR replicates were performed on each of the biological replicates.

**Figure 6:**
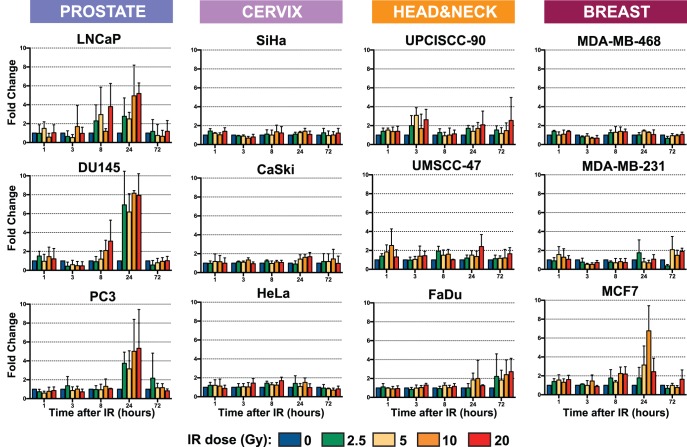
Quantitation of HERV-K expression by qRT-PCR in 12 cancer cell lines after irradiation. Genomic positions of the primers used are shown in Fig. 1. Five different ionizing radiation doses were used (0, 2.5, 5, 10, and 20 Gy), each applied in a single dose, are shown in different colors at five different times following the γ-irradiation (1, 3, 8, 24, and 72 hours). Fold change relative to 0 Gy for each time point was obtained by normalization to 3 housekeeping genes. Bars represent the means of three independent RT-PCR replicates performed for each of three experiments. Error bars show standard deviations.

Most of the cell lines including the three cervical cancer lines tested, the three head and neck tumors, and three of the breast cancer lines showed no notable differences in the level of HERV-K transcripts at any dose of ionizing radiation and at any time point (Fig. 6). However, all three prostate carcinoma cell lines showed significantly increased levels of HERV-K transcripts (Fig. 6). The increases in the three prostate cancer cell lines were most evident at 24 hours following irradiation. The levels of viral *env* RNA at 24 hours were increased two- to eight-fold compared to unirradiated cells. Non-parametric, Wilcoxon-signed rank tests were performed to compare the measurements at each dose of gamma radiation with those at 0 Gy, and the P-values are presented in Table 3. All of the increases in prostate cancer cell lines at 24 hr were significant with P-values <0.01. Thus, they were uniformly significant in these lines and were observed at all radiation doses used. Some increases in these lines were evident in some of the samples at 8 hours post-irradiation, but not at 3 hours. All of the increases were transient, since by 72 hr post-irradiation, the levels of HERV-K had returned to baseline with all doses of γ-irradiation. In general, the highest level of induction was observed at 20 Gy, the highest dose used, although significant increases were observed at all doses and there was no obvious correlation between specific dose and the fold increase among the prostate cancer cell lines.

**Table 3:** P-values for effects of ionizing radiation at 24 hours.

		P-values^a^			
IR dose	cell line	LNCaP	DU145	PC3	MCF7
**0 Gy vs 2.5 Gy**		0.003906	0.003906	0.003906	0.07422
**0 Gy vs 5 Gy**		0.003906	0.003906	0.003906	0.003906
**0 Gy vs 10 Gy**		0.003906	0.003906	0.003906	0.003906
**0 Gy vs 20 Gy**		0.003906	0.003906	0.003906	0.09766

a P-values were obtained using a paired, Wilcoxon-signed rank test on nine replicates for each value.

One of the four breast carcinoma cell lines (MCF-7) also showed an increase in HERV-K RNA levels following γ-irradiation (Fig. 6). The kinetics of the increase were similar to what was observed in the three prostate cancer lines with a maximum increase of six-fold observed 24 hr after irradiation in the 10 Gy treated cells. Both the 5 and 10 Gy doses yielded statistically significant increases (Table 3), and increases of greater than two-fold were observed 8 and 24 hours following several different doses of γ-irradiation, indicating that there was a modest effect of γ-irradiation in this line.

In summary, the levels of HERV-K RNAs were substantially increased by ionizing radiation in a fraction of the cancer cell lines tested. The increases were transient and peaked at about 24 hours post-irradiation. This effect was observed in all three prostate cancer cell lines tested, suggesting that HERV-K in these cells may be particularly sensitive to ionizing irradiation.

## Discussion

The principal findings of this study were that ionizing radiation increased the levels of HERV-K transcripts in some cancer cells, and that the effects varied among the lines tested. In particular, all three prostate cancer lines that were tested exhibited significantly increased levels of HERV-K RNAs, which peaked at about 24 hours post-exposure to a single dose of γ-irradiation and then subsided. A similar increase and subsequent decrease were also observed in one of three breast cancer lines. In the other cancer cell lines tested, the levels of viral RNAs did not appreciably change following γ-irradiation. The mechanisms by which γ-irradiation might alter the levels of HERV-K RNA in lines, particularly prostate cancer lines, are unclear, as is the basis for why it occurred only in certain cell lines. It is curious to note that a small fraction of prostate cancers have translocations of the ETV1 gene (a member of the Ets family of transcription factors) into HERV-K loci [60], [61]. Perhaps HERV-K loci in prostate cells are particularly sensitive to certain unclear phenomena that might affect transcription and other effects. Whatever mechanisms are involved, it took 24 hr to manifest the effects on HERV-K transcript levels, and the levels had returned to baseline by 72 hr (Fig. 6). The cell lines used for this study were chosen because the tumor types that they derived from are frequently treated with γ-irradiation as a component of therapy. The results obtained imply that therapeutic treatment of tumors with ionizing radiation might affect HERV-K RNA levels in some but not all tumors.

The increases in HERV-K RNA levels documented here following γ-irradiation raise at least three potential biological consequences. One is that it may affect immune responses against viral antigens. HERV-K proteins are known to be recognized by immune responses [18]–[20], [22], [23]. Even a transient stimulation in the levels of the viral antigens may provide a window of enhanced susceptibility of such stimulated cancer cells to immune attack, and it may be possible to manipulate these by fractionating the radiation dose. In addition to effects on levels of virus expression, ionizing radiation is known to increase immune responses against cancer cells [14], [62]. We propose that such potential anti-tumor immune effects, along with more extensive analyses of the effects of γ-irradiation on HERV-K in cancer cells, warrant further study.

A second is that it might affect HERV-K proteins that may participate in oncogenesis. Expression of presumably high levels of at least two HERV-K proteins has been shown to be able to contribute to oncogenesis [40], [56]–[58]. γ-irradiation may transiently increase the levels of those proteins in a fraction of tumors.

The third is that it might affect recombination among the HERV-K loci in the human genome. Although no single HERV-K provirus has yet been found in contemporary humans that encodes infectious HERV-K, all the components for viral infectivity exist among the multiple HERV-K proviruses in the human genome today [37]. It is conceivable that double-stranded DNA breaks induced by γ-irradiation may promote recombination among different HERV-K proviruses and generate a single infectious HERV-K genome. Transient increases in HERV-K transcripts such as those observed here might then also involve recombined genomes. The number of mutations that must be corrected to restore infectivity from individual HERV-K components in the human genome today are unknown. Dewannieux et al. [37] engineered three pieces each from a separate provirus to generate an infectious viral genome. In addition, the HERV-K proviruses that existed in the human population in the recent evolutionary past were infectious [37], [38]. Although certain viral mutations such as premature stop codons and frameshifting indels [36], [63] certainly contribute significantly to HERV-K inactivation, the full set of mutations that are responsible for the lack of HERV-K infectivity in humans today is unknown. Such mutations may be as subtle as a conservative single amino acid substitution [64]. Ionizing radiation, like any therapy with mutagenic potential, might occasionally alter those mutations. In addition, the extent of polymorphism of those mutations within the human population is largely unknown. Indeed, it has been suggested recently that HERV-K may be infectious in humans [51]. Future investigations to analyze the effects of ionizing radiation on HERV-K in many types of human tumors would be worthwhile.

This study extended the types of cancer cell lines for which the identification of specific analysis of HERV-K proviruses that encoded the detected transcripts has been undertaken. The key findings were that the specific proviruses varied among the different tumor types and among the specific cell lines derived from them, and that the most recently acquired proviruses in the human genome, those that formed after the human lineage diverged from that leading to chimpanzees, were abundant among those detected. The depth of sequencing employed here limited the detection of transcripts to those that were the relatively most abundant, and it is possible, probably likely, that deeper sequencing would identify additional proviruses. Indeed deeper analysis of HERV-K cDNAs performed by Flockerzi et al. [54] identified essentially the full set of recently acquired HERV-K HML2 proviruses as being transcribed in various tissues. This and other studies that utilized sequencing to identify the loci of origin for HERV-K transcripts emphasize the usefulness and importance of this approach. The proviruses that were identified as being expressed vary substantially in their coding potential from K108 which has full-length open reading frames for all viral proteins [35], [25] to other proviruses, e.g. K(I), that have multiple disruptions to their open reading frames [39]. It is essentially unknown how much those mutations vary within the human population today, and understanding the coding potential of individual proviruses is of importance to harnessing immune responses to HERV-K antigens for practical use. Controls included in these studies to confirm that the RT-PCR products were derived from RNAs and not from contaminating DNA were PCR controls performed without reverse transcriptase and the use of primer pairs that specifically detected spliced RNAs.

The latter led to the frequent detection of alternative splice sites for several specific proviruses, but not for others (Table 2, Fig. 5). This extended previous observations for prostate cancer cell lines [55] into many additional cell lines comprising multiple tumor types. It is likely that the specific sequences of the provirus and thus the transcripts derived from them are important determinants of the use of alternative splice sites. Whether the cellular splicing factor milieu in cancer cells also contributes to the variation observed is unclear. One viral determinant may be the 292 bp deletion that removes the 5′SS for the second intron in doubly spliced viral RNAs (Fig. 1). However, alternative splicing was also detected for the first intron, even though the 292 bp deletion of type 1 proviruses is downstream of the first intron and the splicing signals that immediately flank it (Fig. 5). In addition, K117 is a type 2 provirus, and an alternatively spliced form was detected from it. K117 is 1.3% different from K108, and the latter was uniformly observed to be properly spliced, and one or more of those differences are likely affecting splice site usage. The alternative splice sites detected here were at additional sites than those identified previously [55], suggesting that the choice of sites may be promiscuous. The nucleotide pairs that were situated in the intron immediately downstream of the variant 5′ splice sites were AU, AA, GC, and UU as opposed to the GU at the conventional viral splice site (Figure 5 and [55]). The three nucleotides immediately upstream of the variant 3′ splice sites were AAG, UAG, CAG, GAG, UGG, and ACU (Figure 5 and [55]) as compared with the CAG at the conventional 3′ splice site for the first intron of HERV-K. Thus the sequences of most of the alternative sites that were detected shared partial overlap with those of the conventional splicing signals. These various results raise the question of what determinants within the viral genome affect the utilization of alternative splice sites, and further studies of HERV-K may shed light on the basic mechanisms of splicing.

In summary, the effects of ionizing radiation on HERV-K as well as the alternative splicing indicate that further study of these phenomena are warranted. HERV-K has been reported to be expressed in many types of human tumors [55], [65]–[71] and these studies also indicate that a far more extensive analysis of the effects of ionizing radiation on HERV-K including many more types of tumors and primary tissue as well as cell lines are worth pursuing.

## Methods

### Cell Lines and Culture Conditions

Three human prostate cancer cell lines (LNCaP, DU145, and PC3), four cervix cancer cell lines (C33A, SiHa, CaSki, and HeLa), one head and neck cancer cell line (FaDu) and three breast cancer cell lines (MDA-MB-468, MDA-MB-231, and MCF7) were purchased from ATCC (Manassas, VA, USA). UM-SCC-47 and UPCI-SCC-90 [72] cell lines were obtained from Dr. Douglas Trask (University of Iowa) and Dr. Suzanne Gollin (University of Pittsburgh Cancer Institute), respectively. Cell lines were grown in 100 mm petri dishes in 5% CO_2_ in a humidified 37°C incubator to 90% confluence.

### Irradiation Conditions

From each cell line, subsets at 50% confluence were treated with γ-irradiation (J.L. Shepherd Mark I ^137^Cs Irradiator) at 0, 2.5, 5, 10 or 20 Gy administered in a single dose. For each cell line, one subset was harvested for each of five timepoints (1, 3, 8, 24, and 72 hours after irradiation). A total of three independent experiments were performed for each cell line.

### RNA Extraction

Total RNA was extracted using Trizol (Invitrogen). RNA was then subjected to DNAse I digestion (TURBO DNA-free kit - Applied Biosystems #AM1907) to remove any genomic DNA contamination. RNA quality and concentration were determined by evaluation of rRNA bands in agarose gel electrophoresis and by NanoDrop spectrophotometric analysis (Thermo Scientific).

### RT-PCR and Sequencing

To identify the specific transcriptionally active HERV-K loci, 3 µg of each RNA was used for RT-PCR. Primers were designed in well-conserved segments of HERV-K genome to ensure amplification of as many loci as possible. A set of primers was designed across the viral *env* splicing junction to identify the singly and doubly spliced variants of HERV-K transcript. Parallel controls were performed to detect GAPDH transcripts. Primer sequences were as follows:

1X-env(1)-Fwd: 5′-AGGGAAAAACCGCCTTAGGG-3′

1X-env(2)-Fwd: 5′-TGCGGGCAGCAATACTGCT-3′

1X-env-Rev: 5′-CACCGCACTATTGGCCACA-3′

2X-rec(1)-Fwd: 5′-AGGGAAAAACCGCCTTAGGG-3′

2X-rec(2)-Fwd: 5′-CAGATGAAGTTGCCATCCACCA-3′

2X-rec-Rev: 5′-ACAAAACCGCCATCGTCATC-3′

2X-np9-Fwd: 5′-GGAGATGCAAAGAAAAGGGCCT-3′

2X-np9-Rev: 5′-ACAAAACCGCCATCGTCAT-3′

pro-Fwd: 5′-AAACGAGCAAAGGGGCCA-3′

pro-Rev: 5′-TTTCCCTAGTCCCTTTCCTGGT-3′

pol-Fwd: 5′-CTGGTGCATGGAAGATTGGT-3′

pol-Rev: 5′-ACAAGCAAAACCTCTCCCCCA-3′

env(1)-Fwd: 5′-CATGGTAAGCGGGATGTCACT-3′

env(2)-Fwd: 5′-ATGTCACTCAGGCCACGG-3′

env-Rev: 5′-ACAAAACCGCCATCGTCATC-3′

hGAPDH-Fwd: 5′-AGATCATCAGCAATGCCTCCT-3′

hGAPDH-Rev: 5′-AGTCTTCTGGGTGGCAGTG-3′

The reverse transcription reactions (SuperScript III First-Strand Synthesis System - Invitrogen #18080-051) were performed using 3 µg RNA in an initial volume of 10 µL with 1 µL of dNTP mix 10 mM, Oligo(dT)_20_ primer for a final concentration of 2.5 µM and DEPC-treated water. After an initial denaturation step, 5 minutes at 65°C followed by 1 min at 4°C, 2 µL of 10X RT buffer, 4 µL of 25 mM MgCl_2_, 2 µL of 0.1 M DTT, 40 U RNaseOUT, and 200 U SuperScript III RT enzyme were added to reach a final volume of 20 µL. The RT elongation step was performed at 50°C for 50 minutes, followed by enzyme heat inactivation at 85°C for 5 minutes. After brief cooling of the sample at 4°C, digestion of residual RNA was performed with 2 U RNAse-H at 37°C for 20 minutes. Parallel experiments in which no RT enzyme was added were simultaneously carried out.

PCR was performed with 4 µL of RT product, 200 nM primers 200, Platinum PCR SuperMix HiFi (Invitrogen) and nuclease-free water to a total volume of 20 µL. After a denaturation step at 94°C for 2 minutes, 30 cycles of denaturation-annealing-elongation were performed, followed by final elongation at 68°C for 7 minutes. Denaturation was performed at 94°C for 25 seconds, annealing for 25 seconds, and extension at 68°C for each experiment as follows: Detection of pro, pol and env amplicons (Fig. 2) were performed with annealing temperature of 58°C followed by extension time of 1.5 minutes; 2 µL of a 1∶200 dilution of the PCR product was then used in nested PCR reaction for env with annealing temperature of 61°C followed by extension time of 1.5 minutes (Fig. 3). Detection of 1X-env amplicon (Fig. 2) was performed with a reaction at annealing temperature of 58°C followed by extension time of 2 minutes; 2 µL of a 1∶200 dilution of the PCR product was then used in nested PCR reaction with annealing temperature of 61°C followed by extension time of 2 minutes (Fig. 3). Detection of 2X-env amplicon (Fig. 2) was performed with a reaction at annealing temperature of 58°C followed by extension time of 1 minutes; 2 µL of a 1∶200 dilution of the PCR product was then used in nested PCR reaction with annealing temperature of 61°C followed by extension time of 1 minute (Fig. 3).

Electrophoresis of PCR products was performed in 1% agarose gels. PCR products obtained were purified (PCR purification - Qiagen), and recovered cDNA was sequenced. For shotgun subcloning analyses, PCR products were cloned (TOPO TA cloning - Invitrogen) into pCR4 plasmids using TOP10-DH5α competent cells and sequenced.

### Identification of HERV-K Loci

Each sequence obtained, trimmed of any vector and primers sequences, was aligned to the human genome using BLAT software [73]. The sequence was assigned to the locus obtaining the highest identity score (Tables 1 and 2).

### Quantitative RT-PCR (qRT-PCR)

To quantify HERV-K transcripts, 1 µg of RNA from each set was used for two-step, qRT-PCR (Superscript III first strand - Invitrogen) and PowerSybr Green (AppliedBiosystems). RT (SuperScript III First-Strand Synthesis System - Invitrogen #18080-051) of 1 µg RNA was performed in an initial volume of 10 µL with 1 µL of 10 mM dNTP mix, 1 µL of 50 µM Oligo(dT)_20_ primer, and DEPC-treated water. After an initial denaturation step of 5 minutes at 65°C followed by 1 min at 4°C, 2 µL of 10X RT buffer, 4 µL of 25 mM MgCl_2_, 2 µL of 0.1 M DTT, 40 U RNaseOUT, and 200 U SuperScript III RT enzyme were added to reach a final volume of 20 µL. The RT elongation step was performed at 50°C for 50 minutes, followed by enzyme heat inactivation at 85°C for 5 minutes. After brief cooling of the sample at 4°C, digestion of residual RNA was performed with 2 U RNAse-H at 37°C for 20 minutes. Parallel experiments with no RT enzyme were carried out simultaneously.

1 µL of a 1∶10 dilution of the RT product was then added to 2x SYBR Green PCR Master Mix (AppliedBiosystems #4367659) and nuclease-free water to a final volume of 8 µL. Primers q-env-Fwd and q-env-Rev were designed in a well-conserved region of HERV-K genome common to unspliced and once-spliced transcript variants downstream of the Δ292 deletion characteristic of type-I HERV-Ks. Four housekeeping genes, human GAPDH (hGAPDH), human beta glucuronidase (hGUSB), human hydroxymethybilane synthase (hHMBS), and human phosphoglycerate kinase 1 (hPGK1) were used as endogenous controls for normalization. Primer sequences as follows:

q-env-Fwd: 5′-TCACATGGTAAGCGGGATGTC-3′

q-env-Rev: 5′-CGCACTATTGGCCACACATTC-3′

hGAPDH-Fwd: 5′-AGATCATCAGCAATGCCTCCT-3′

hGAPDH-Rev: 5′-AGTCTTCTGGGTGGCAGTG-3′

hGUSB-Fwd: 5′-CGCGCCGACTTCTCTGACAA-3′

hGUSB-Rev: 5′-CCACACCCAGCCGACAAAA-3′

hHMBS-Fwd: 5′-CTTCCTCCTGGCTTCACCAT-3′

hHMBS-Rev: 5′-GGTTCCCACCACACTCTTCT-3′

hPGK1-Fwd: 5′-AGCGGGTCGTTATGAGAGT-3′

hPGK1-Rev: 5′-ACTACCGACTTGGCTCCAT-3′

Three replicates per sample were used in 384-well plate PCR system (7900HT - AppliedBiosystems) with cycling conditions of 50°C for 2 minutes, 95°C for 10 minutes, 40 cycles of 95°C for 10 seconds, 60°C for 20 seconds and 72°C for 30 seconds, and three biological replicates were performed. Data analysis was performed with SDS 2.4 and DataAssist softwares to determine means and standard deviations and to normalize the levels of viral transcripts to those of the housekeeping genes (AppliedBiosystems).
